# Meta-analysis of diagnostic accuracy studies in mental health

**DOI:** 10.1136/eb-2015-102228

**Published:** 2015-10-07

**Authors:** Yemisi Takwoingi, Richard D Riley, Jonathan J Deeks

**Affiliations:** 1 Public Health, Epidemiology and Biostatistics, University of Birmingham, Birmingham, UK; 2 Research Institute for Primary Care and Health Sciences, Keele University, Staffordshire, UK

## Abstract

**Objectives:**

To explain methods for data synthesis of evidence from diagnostic test accuracy (DTA) studies, and to illustrate different types of analyses that may be performed in a DTA systematic review.

**Methods:**

We described properties of meta-analytic methods for quantitative synthesis of evidence. We used a DTA review comparing the accuracy of three screening questionnaires for bipolar disorder to illustrate application of the methods for each type of analysis.

**Results:**

The discriminatory ability of a test is commonly expressed in terms of sensitivity (proportion of those with the condition who test positive) and specificity (proportion of those without the condition who test negative). There is a trade-off between sensitivity and specificity, as an increasing threshold for defining test positivity will decrease sensitivity and increase specificity. Methods recommended for meta-analysis of DTA studies --such as the bivariate or hierarchical summary receiver operating characteristic (HSROC) model --jointly summarise sensitivity and specificity while taking into account this threshold effect, as well as allowing for between study differences in test performance beyond what would be expected by chance. The bivariate model focuses on estimation of a summary sensitivity and specificity at a common threshold while the HSROC model focuses on the estimation of a summary curve from studies that have used different thresholds.

**Conclusions:**

Meta-analyses of diagnostic accuracy studies can provide answers to important clinical questions. We hope this article will provide clinicians with sufficient understanding of the terminology and methods to aid interpretation of systematic reviews and facilitate better patient care.

## Introduction

Diagnostic accuracy is the ability of a test to correctly identify or exclude a target condition and is a fundamental part of the evaluation of medical tests. Test accuracy is estimated by comparing results of an index test (a new or existing test of interest) with a reference standard, sometimes known as a ‘gold’ standard, to give a 2×2 table of the number of true positives, false positives, false negatives and true negatives (table 1). The reference standard is used to verify the presence or absence of the target condition, and may be a single test or a combination of tests.^1^ The ideal index test should have no misclassification errors (false negatives or false positives), but in clinical practice such a test is unlikely to exist.

**Table 1:** Cross classification of index test and reference standard results

	Reference standard positive	Reference standard negative	Total
Index test positive	a (true positives)	b (false positives)	a+b (test positives)
Index test negative	c (false negatives)	d (true negatives)	c+d (test negatives)
Total	a+c (disease positives)	b+d (disease negatives)	a+b+c+d (study total)

Many test accuracy studies are small,^2^ and even when studies are large, the number of cases may be small due to the low prevalence of the target condition. A systematic review and meta-analysis of diagnostic test accuracy (DTA) aims to identify and summarise evidence on the accuracy of tests, including an assessment of the quality and consistency of the evidence. Pooling the results of multiple studies addressing the same question using meta-analysis will provide a more precise estimate of test performance than is possible in a single study. The extent of variability in test performance between studies (heterogeneity) can be quantified in a meta-analysis and formal investigations of potential sources of heterogeneity may also be performed in order to explain why results differ between studies.

The rate of publication of systematic reviews and meta-analyses of diagnostic accuracy has risen considerably since the 1990s,^3^ and they are being used to inform evidence-based clinical practice guidelines and healthcare policy.^4–7^ Producing DTA reviews is more complex than reviews of interventions.^8^ ^9^ Meta-analysis is one of the challenging aspects because traditional meta-analytic methods used for intervention reviews are not appropriate for DTA reviews. It is vital that recommended methods for pooling study results are well understood to ensure appropriate application. In this paper, we summarise basic concepts in diagnostic accuracy research as a prelude to explaining the rationale for the methods recommended for DTA meta-analysis, describe the properties of the methods, and use a published example to illustrate their application in different types of analyses. We focus on commonly used methods for situations where a single 2×2 table is available, or can be derived for each study included in a meta-analysis.

## Basic concepts in diagnostic accuracy research

It is important to be aware of both the types of data that tests produce and the statistical measures used to summarise test accuracy.

### Types of data

Test results may be expressed as measurements (counts or continuous) or classifications (binary or ordered categories). Standard methods for computing test accuracy demand binary classification of the results of the index test and the reference standard (table 1). As such, for non-binary data, criteria for determining test positivity, typically referred to as thresholds, cut-offs or cut points, are needed to dichotomise the data. Thresholds may be explicit numeric values or implicit based on different criteria derived from subjective visual interpretation or measurements. For example, various numeric thresholds have been used to define a positive result for the Informant Questionnaire on Cognitive Decline in the Elderly (IQCODE) for detection of dementia.^10^ Higher scores are indicative of greater cognitive impairment, with a maximum average score of 5. In a Cochrane DTA review, Quinn *et al*^10^ described the test accuracy of the 16-item and 26-item IQCODE for thresholds of 3.3, 3.4, 3.5 and 3.6.

### Types of measures of test accuracy

The most commonly used measures are summarised in table 2. Measures are either paired or single (global) indicators of test performance. The paired measures—sensitivity and specificity, positive and negative predictive values, and positive and negative likelihood ratios—separately describe the performance of a test for ascertaining first the presence and then the absence of the target condition. Sensitivity and specificity are the most commonly reported measures in primary studies and are also used for meta-analysis.^3^ A receiver operating characteristic (ROC) plot is used to show how as the test threshold decreases (for a test where the presence of disease increases the value of the test measurement such as the IQCODE example above) sensitivity increases while specificity decreases, and vice versa.^11^ The position of the ROC curve depends on the discriminatory ability of the test; the more accurate the test is, the closer the curve to the upper left hand corner of the ROC plot. A test that performed no better than tossing a coin would have an ROC curve along the 45° axis.

**Table 2:** Definition of common measures of test accuracy

Test accuracy measure	Formula*	Definition
Sensitivity	a/(a+c)	Proportion of those with the target condition correctly identified as having the condition
Specificity	d/(b+d)	Proportion of those without the target condition correctly identified as not having the condition
Positive predictive value	a/(a+b)	Proportion of those with the target condition out of the test positives
Negative predictive value	d/(c+d)	Proportion of those without the target condition out of the test negatives
Positive likelihood ratio (LR^+^)	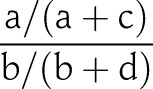	Ratio of the proportion that tests positive among those with the target condition compared to the proportion that tests positive among those without the target condition
Negative likelihood ratio (LR^–^)	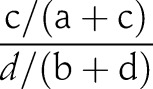	Ratio of the proportion that tests negative among those with the target condition compared to the proportion that tests negative among those without the target condition
Diagnostic OR	ad/bc or LR^+^/LR^–^	Ratio of the odds of positivity in those who have the target condition relative to the odds of positivity in those without the condition

*Expressed based on the notation used in table 1.

The most common global measures are the diagnostic OR (DOR) and the area under the curve (AUC). These measures summarise the accuracy of the test across all possible thresholds, but are not helpful in clinical practice because they do not provide information on the error rates in the diseased (false negative) and non-diseased groups (false positives). The error rates are important for judging the extent and likely impact of the downstream consequences of testing. In meta-analysis, the DOR can be a useful measure when comparing tests or subgroups, particularly if there is no preference for either superior sensitivity or specificity, and interest is in global performance.

## Types of analyses in a diagnostic accuracy review

Three main types of analyses can be performed in a DTA review reflecting the types of questions and objectives that can be addressed.^11^ These are:

1. Analysis of a single test—What is the diagnostic accuracy of the test?
2. Analysis comparing multiple tests—How does the accuracy of two or more tests differ?
3. Investigation of heterogeneity—How does test accuracy vary with clinical and methodological characteristics?

Each of these will be considered separately below, using an example. First, the broad principles of the meta-analysis approaches are discussed.

## Methods for meta-analysis: broad principles

We recommend first plotting the data—the results of each study can be marked as a sensitivity and specificity point in a summary ROC (SROC) plot which will depict the location of the data (consider how close the points lie to the 100% sensitivity and 100% specificity lines); the scatter of the points; and any relationship between sensitivity and specificity obvious across multiple studies. Usually only one point is plotted per study, but see the multiple thresholds discussion later. Since the different studies included in a meta-analysis may explicitly use different thresholds, or variations in the way the test is interpreted and applied, and even the patient group may induce threshold-like differences between studies, the recommended meta-analytical methods for test accuracy all explicitly or implicitly allow for the negative correlation between sensitivity and specificity across studies that is induced by the threshold-like variation. Simple univariate meta-analytic methods pool sensitivity and specificity separately, ignoring the potential threshold effect. Such analyses can give misleading results as illustrated by Irwig *et al*.^12^ An SROC curve approach was developed by Moses *et al*^13^ to account for possible heterogeneity in threshold, but the approach assumes that variation is only due to the threshold effect and chance and does not allow for heterogeneity. Measures of test accuracy are not fixed properties of a test and often vary with the population, setting, characteristics and conduct of the test (including skill and experience of assessors or practitioners), and definition of the target condition. Therefore, heterogeneity is a common feature of DTA reviews. The Moses SROC approach also has other important methodological limitations^14^ ^15^ and should only be used for preliminary analyses.^11^

Special hierarchical models have been developed for DTA meta-analysis that account for the negative correlation in paired measures across studies and heterogeneity. These models are termed hierarchical models because they involve statistical distributions at two levels; within-study variability in sensitivity and specificity is taken into account at the lower level, and between-study variability at the higher level.^11^ The bivariate^16^ ^17^ and the hierarchical summary receiver operating characteristic (HSROC) models^18^ are the two hierarchical models used for meta-analysis when a single sensitivity and specificity pair is available for each study.^8^ ^11^

The focus of the bivariate model is estimation of a summary point (summary sensitivity and specificity) at a common threshold. This would be useful if studies used a standard threshold and clinicians wanted to know how the test performed at that threshold (eg, the Mini-Mental State Examination at a threshold of 24/30 indicating normal). In contrast, the focus of the HSROC model is on estimating an SROC curve across different thresholds (note this is not the same curve as the Moses SROC curve). This would be useful if studies used a variety of thresholds and clinicians wanted to get an idea of how the test performed across the range of thresholds, or wanted to compare tests without restricting comparison to a single threshold. Ideally, we would like to know which threshold on the curve gives the best performance, but the position of individual thresholds cannot be identified. The bivariate and HSROC models have been shown to share statistical properties.^19^ The choice of which model to use should ideally be driven by the research question (ie, focus of inference on points or curves) but is sometimes influenced by the nature of the available data (mixed thresholds) and its effect on the interpretation of summary findings, software capability and expertise of the team. Owing to their shared statistical properties, SROC curves can be computed from bivariate models and average operating points from HSROC models, so the choice of model when there is only a single test is academic. When there are comparisons between tests or subgroups, the choice of model is important.

Bivariate meta-analysis of likelihood ratios and predictive values are alternatives to bivariate meta-analysis of sensitivity and specificity but have been noted to face additional challenges.^20^ ^21^ Therefore, only methods for summarising sensitivities and specificities are illustrated in this paper. Other measures such as likelihood ratios can be derived from functions of some of the parameters of bivariate or HSROC models shown in table 3. The example below is used to illustrate different types of analyses and the appropriate use of hierarchical models.

**Table 3:** Basic parameters of bivariate and HSROC models

HSROC model	Bivariate model
Mean accuracy	Mean logit sensitivity
Mean threshold	Mean logit specificity
Variance of random effects for accuracy	Variance of random effects for logit sensitivity
Variance of random effects for threshold	Variance of random effects for logit specificity
Shape of SROC curve	Correlation between the logits of sensitivity and logits of specificity

Each of the models has five parameters when no covariates are included.

HSROC, hierarchical summary receiver operating characteristic; SROC, summary ROC.

## Working example: screening tests for bipolar spectrum disorders

**Figure 1:**
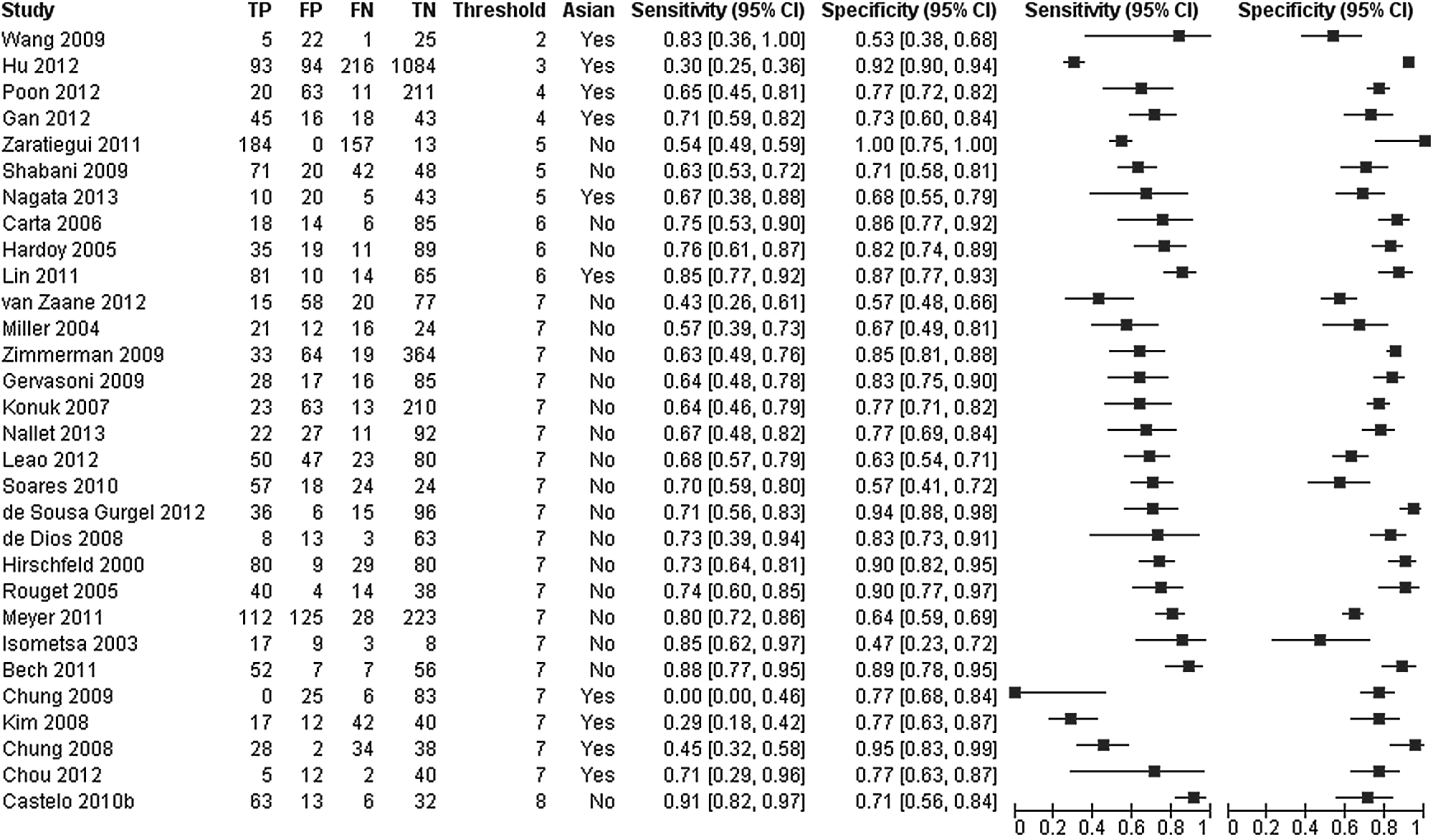
Forest plot of sensitivity and specificity of the MDQ for detection of any type of bipolar disorder in mental health centre settings . Two covariates (threshold and language of the instrument) are shown on the plot. The studies are ordered according to the threshold, language of the instrument (Asian, yes or no) and sensitivity (FN, false negative; FP, false positive; MDQ, mood disorder questionnaire; TN, true negative; TP, true positive).

A DTA systematic review compared the diagnostic accuracy of three screening questionnaires—the bipolar spectrum diagnostic scale (BSDS), the hypomania checklist (HCL-32) and the mood disorder questionnaire (MDQ)—for detection of bipolar disorder (table 4).^22^ Studies used different thresholds to define test positivity for each instrument. The reference standards used were based on the Diagnostic and Statistical Manual of Mental Disorders 4th Edition (DSM-IV) criteria. The review included analyses for different settings and types of bipolar disorder. Only the analysis of bipolar disorder in general in a mental health centre setting is considered here (figure 1).

**Table 4:** Characteristics of the index tests in the example systematic review

Characteristic	Index tests		
	Bipolar spectrum diagnostic scale	Hypomania checklist	Mood disorder questionnaire
Number of studies	8	17	30
Range of total score	0–25 points	0–32 points	0–15 points
Recommended thresholds*	13	14	7

*Thresholds recommended by the developers of each screening instrument.^32–34^

## Methods for meta-analysis of a single test

### Data synthesis

#### Estimation of a summary sensitivity and specificity at a fixed threshold

The bivariate model jointly synthesises sensitivity and specificity to give summary estimates which are drawn as the summary point on an SROC plot. Confidence and prediction regions plotted around the summary point enable joint inferences to be made about sensitivity and specificity. These regions account for correlation between sensitivity and specificity, and are useful for illustrating uncertainty around the summary point and the extent of heterogeneity. Since summary points should only be calculated when studies share a common threshold, the available data are reduced. The choice of a common threshold is often based on the available data and may not be the threshold used in clinical practice. Furthermore, a common threshold is difficult to define for non-numeric tests.

**Figure 2:**
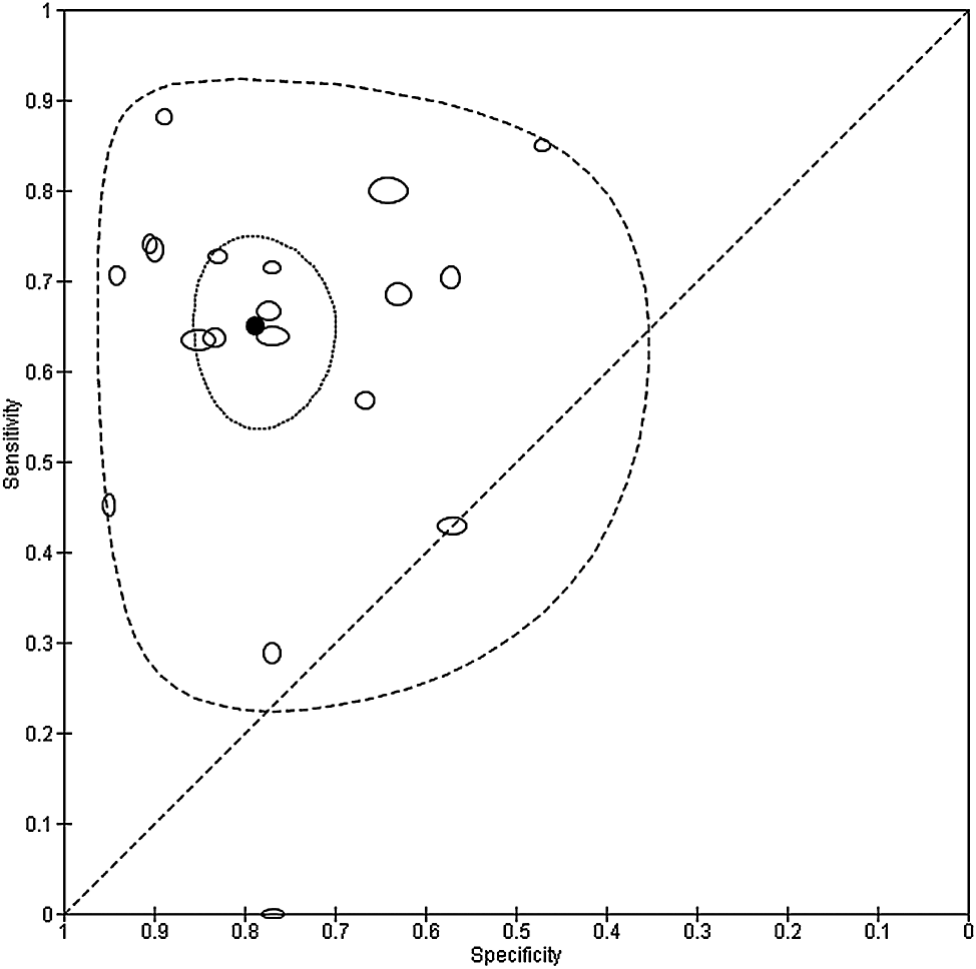
Summary receiver operating characteristic plot of the mood disorder questionnaire (MDQ) at a common threshold of 7 for detection of any type of bipolar disorder in mental health centre settings. The size of each point is scaled according to the precision of sensitivity and specificity for the study. The solid circle (summary point) represents the summary estimate of sensitivity and specificity for the MDQ at a threshold of 7. The summary point is surrounded by a dotted line representing the 95% confidence region and a dashed line representing the 95% prediction region (the region within which we are 95% certain that the results of a new study will lie).

The summary point for the MDQ can be estimated by meta-analysis of only studies that used the recommended threshold of 7. This restriction reduces the data for meta-analysis from 30 to 19 studies. The summary sensitivity and specificity were 0.65 (0.57 to 0.72) and 0.79 (0.72 to 0.84), respectively. Figure 2 shows this summary point with a 95% confidence region and 95% prediction region. The confidence region is based on the CI around the summery point and indicates that, on the basis of the available data, we would expect the ‘real value’ to be within that region 95% of the time. The prediction region around the summary point indicates the region where we would expect results from a new study in the future to lie, and is therefore wider than the confidence region as it goes beyond the uncertainty in the available data. Despite the use of a common threshold, considerable heterogeneity is evident from the plot.

#### Estimation of a summary curve

The HSROC model focuses on estimation of an SROC curve. The advantage of this approach is that data from each study can be included irrespective of the threshold used, thus maximising use of the available data. Note that only one 2×2 table per study is included in a meta-analysis, and therefore a choice needs to be made for studies that report multiple thresholds.^11^ Although a summary point on an SROC curve estimated using mixed thresholds is clinically uninterpretable, estimates of sensitivity and their CIs can be computed from the HSROC model at fixed values (eg, lower quartile, median and upper quartile) of specificity, or vice versa, to illustrate changes in sensitivity and specificity along the curve.

**Figure 3:**
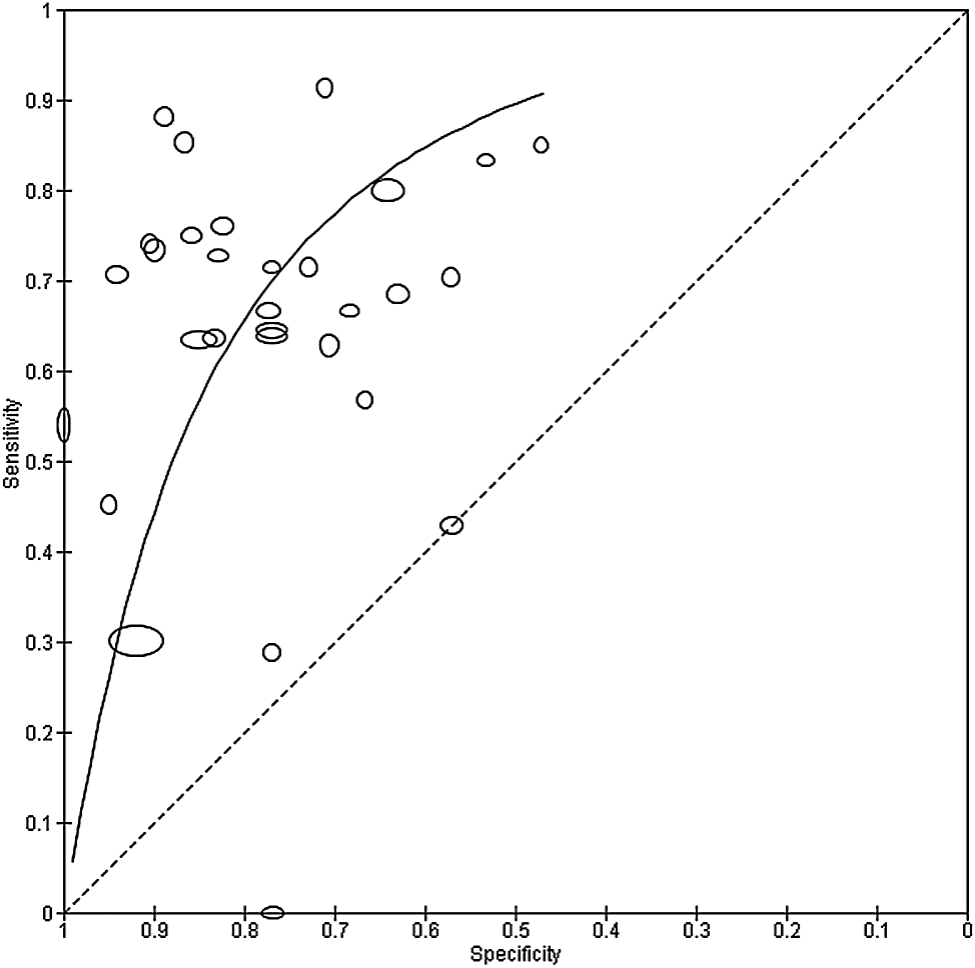
Summary receiver operating characteristic plot of the mood disorder questionnaire (MDQ) at different thresholds for detection of any type of bipolar disorder in mental health centre settings. Each study point was scaled according to the precision of sensitivity and specificity for the study. The summary curve was drawn restricted to the range of specificities (0.47 to 1.00) from the 30 studies included in the evaluation of the MDQ.

Figure 3 shows the summary curve for the MDQ. To avoid extrapolation beyond the data, the curve was drawn within the range of observed specificities (0.47 to 1.00) from the 30 included studies. For illustration of a point on the curve, the estimated sensitivity was 0.70 (0.64 to 0.77) at the median specificity of 0.77 from the included studies. Given the relationship between the bivariate and HSROC models mentioned earlier,^19^ it is possible to estimate summary sensitivity and specificity by estimating an average operating point on the SROC curve. This analysis can be performed separately for studies that report data at a common threshold. For instance, the summary sensitivity and specificity from the HSROC method restricted to studies with threshold 7 are the same as those obtained from the bivariate model in the preceding section.

### Measuring and explaining heterogeneity

The traditional I^2^ statistic^23^ is not recommended for quantifying heterogeneity in sensitivity and specificity because it is a univariate measure that does not account for potential threshold effects. To investigate whether a factor is associated with test accuracy, exploratory analyses can be performed by visual inspection of forest plots and SROC plots. Where formal investigations are possible, meta-regression can be performed by adding the factor as a covariate to a hierarchical model. If there are mixed thresholds, the analysis based on an HSROC model fitted to all relevant studies will have more power than an analysis at a fixed threshold. Owing to the complexity of hierarchical models and paucity of data, it is usual to assess the effect of covariates one at a time.

The bivariate model allows covariates to affect sensitivity, specificity or both. The HSROC model allows covariates to affect the accuracy, threshold and/or shape of the SROC curve. If SROC curves are assumed to have the same shape (ie, parallel curves), differences in test performance can be expressed as the relative diagnostic OR (rDORs) comparing the DOR of one group of studies to that of another. The fit of alternative models (effect of adding or removing covariate terms from the bivariate or HSROC model) can be assessed using statistical tests to compare models with and without covariate terms. Covariates to be investigated should be prespecified with a clear justification for their selection.

**Figure 4:**
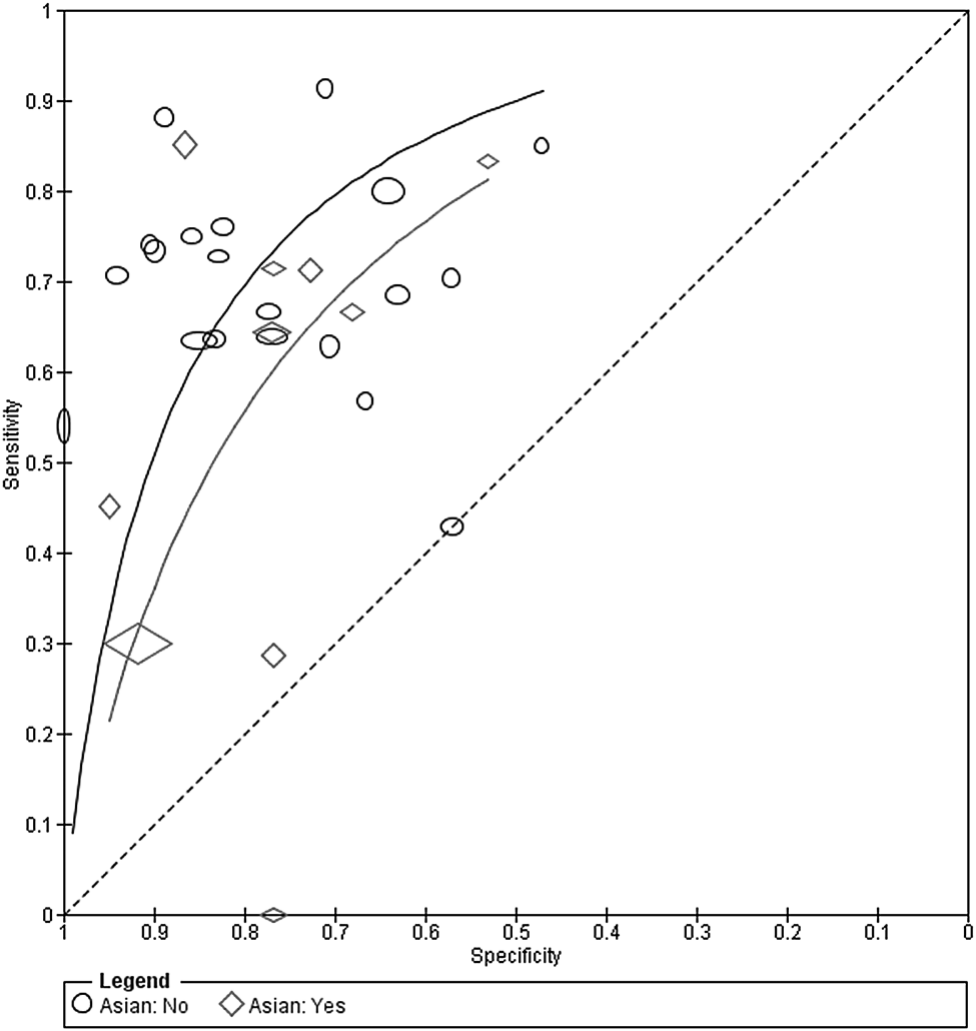
Summary receiver operating characteristic curves comparing Asian and non-Asian language versions of the mood disorder questionnaire. Each study point was scaled according to the precision of sensitivity and specificity for the study. The summary curves were drawn restricted to the range of specificities for each group of studies (0.47 to 1.00 for non-Asian and 0.53 to 0.95 for Asian studies).

The effect of language (Asian vs non-Asian) on the accuracy of the MDQ was assessed by comparing SROC curves for the two subgroups of the covariate in one HSROC model (figure 4). Ten studies used an Asian language version while 20 studies used a non-Asian language version of the MDQ (figure 1). The model was fitted by including covariate terms for accuracy and threshold, but the curves were assumed to have the same shape. The estimated DOR (95% CI) of Asian and non-Asian language versions were 5.01 (2.64 to 9.51) and 9.14 (5.90 to 14.1), respectively. The rDOR (95% CI) of 0.55 (0.25 to 1.19) indicates that the DOR of Asian versions was 0.55 times that of the non-Asian versions, though there was no statistical evidence of a difference in accuracy (p=0.13).

## Methods for comparisons of multiple tests

Most DTA reviews only evaluate the accuracy of a single test, yet reviews comparing the accuracy of two or more tests are likely to be of greater relevance to clinicians and policymakers where decisions need to be made about selecting tests for use in practice. Different analytic strategies and meta-analytic models can be used for test comparisons. These are explained below.

### Test comparison strategy

Ideally, test comparisons should focus on studies that have directly compared the index tests evaluated in the review.^8^ ^24^ Direct comparisons are likely to ensure an unbiased comparison, but such analyses are not always feasible due to the limited availability of comparative studies.^24^ An indirect comparison uses all eligible studies that have evaluated at least one of the tests of interest, thus maximising use of the available data. However, the difference in accuracy is prone to confounding due to differences in patient and study characteristics. A comparative review may include indirect, direct or both types of comparisons.

### Comparison of summary points or SROC curves

**Figure 5:**
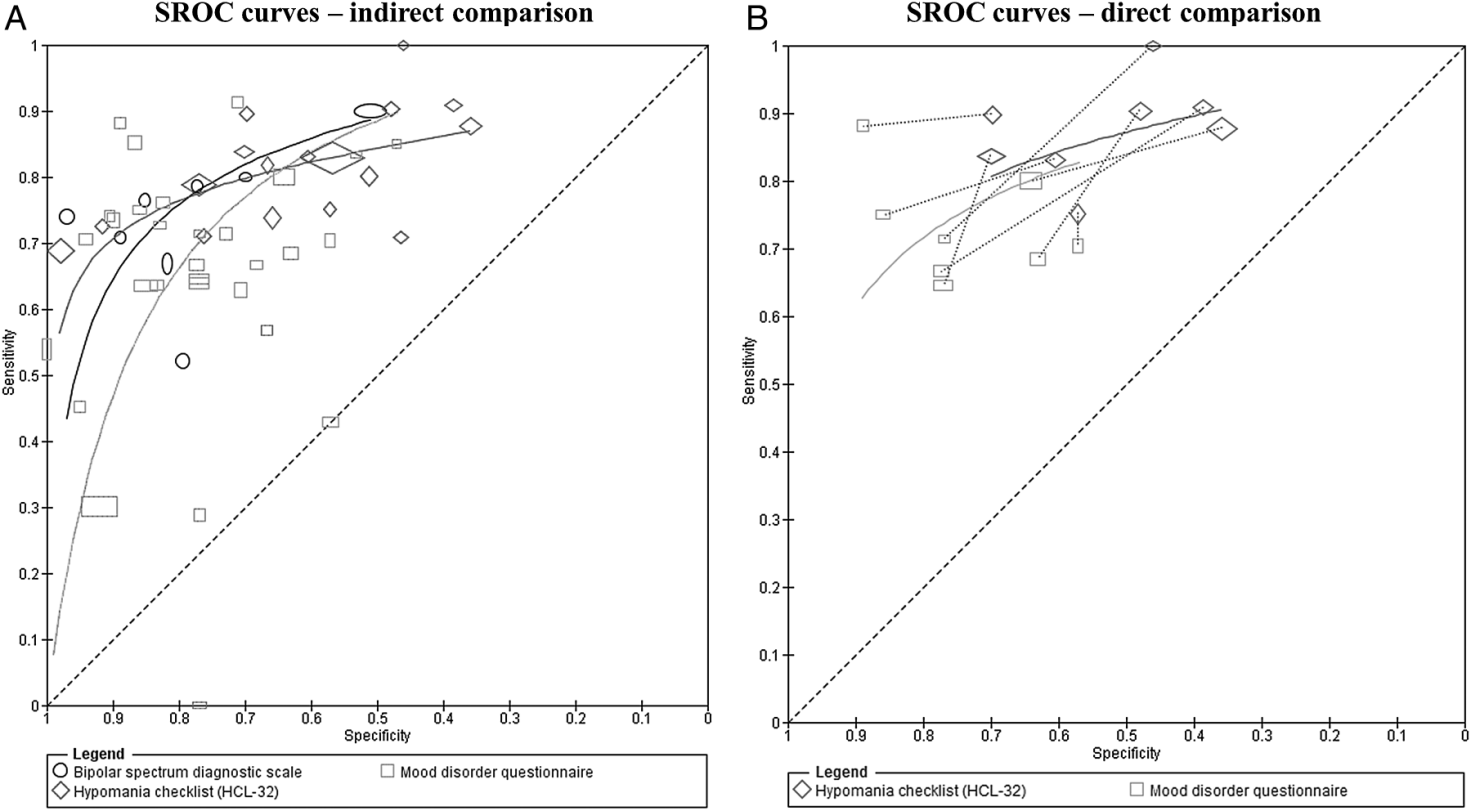
SROC plot comparing the accuracy of BSDS, HCL-32 and MDQ for detection of any type of bipolar disorder in mental health centre settings. For each test on an SROC plot, each symbol represents the pair of sensitivity and specificity from a study. The study points were scaled according to the precision of sensitivity and specificity in the studies. Each SROC curve was drawn restricted to the range of specificities from included studies that evaluated the test. The SROC plot in panel (A) is an indirect comparison (includes all studies that evaluated any of the tests) while panel (B) is a direct comparison where analysis were restricted to only studies that compared both tests in the same patients. A line connects the pair of points representing the two tests from each study (adapted from Carvalho *et al*^22^; BSDS, bipolar spectrum diagnostic scale; HCL-32, hypomanic checklist; MDQ, mood disorder questionnaire; SROC, Summary receiver operating characteristic). Reprinted from ref 22 with permission from Elsevier.

Test comparisons may be based on a comparison of summary points and/or SROC curves. Although points can be estimated at different common thresholds for each test, the ranking of tests may not be consistent at different thresholds, and therefore a comparison of curves may be more appropriate in such situations. This is evident in figure 5A which shows that the SROC curves for the three tests cross, indicating that no test is consistently more accurate than any of the others and relative accuracy depends on the choice of threshold. Current methods recommended for meta-analysis of test comparisons use a meta-regression approach, by including test type as a covariate in a hierarchical model to assess differences in test accuracy.^11^ This is the same as the meta-regression approach described earlier for investigation of heterogeneity. Covariate terms can be allowed to depend on any of the model parameters shown in table 3, including the variance parameters. For an example of a comparison of summary points, see Abba *et al*.^25^ A comparison of SROC curves is illustrated below.

### Comparison of the MDQ, BSDS and HCL-32

The diagnostic accuracy of the three tests was compared using an HSROC meta-regression model. For a full description of the methods and interpretation of the findings, see Carvalho *et al*.^22^ All 44 studies of the three tests were included in an indirect comparison (figure 5A). There was statistical evidence that the shape of the SROC curves differed, implying that the relative accuracy of the tests varies with thresholds. For the direct comparisons, eight studies compared the MDQ and HCL-32 (figure 5B panel B), and three studies compared the BSDS and MDQ. None of the 44 studies directly compared the HCL-32 and BSDS. Although the studies used different thresholds, the results from the eight studies were consistent with the HCL-32 showing higher sensitivity and lower specificity than the MDQ as shown in figure 5B. The connecting line between a pair of points identifies the results for the pair of tests from each study. A meta-analysis was performed comparing the MDQ and HCL-32. There was no evidence of a difference in accuracy.

## Software options and extensions

The statistical packages available for fitting the HSROC model are currently limited to SAS, WinBUGS and R. These packages can also be used to fit the bivariate model, as can Stata and MLWin. Review Manager, the Cochrane Collaboration's review authoring tool, was used to produce the forest plot and SROC plots shown in this paper. Traditionally, the SROC plot is a plot of sensitivity against 1–specificity but because interest is often in sensitivity and specificity, Review Manager plots sensitivity against specificity on a reversed scale. Review Manager cannot be used to fit either hierarchical model but is useful for exploratory analyses, and for producing forest plots and SROC plots using parameters from hierarchical models fitted using one of the statistical packages. SAS programs for fitting hierarchical models for analysis of a single test, test comparisons and investigations of heterogeneity are available in the Cochrane Handbook for DTA reviews.^11^ An SAS macro^26^ that makes fitting the models more accessible and a tutorial describing how to use RevMan with Stata for different types of analyses is available at http://dta.cochrane.org/. Other user written programs are available for Stata (eg, metandi, midas) and R (eg, bamdit, DiagMeta, mada, HSROC). Extensions to the hierarchical methods discussed are emerging, in particular to deal with multiple thresholds from the same study, which require more complex modelling of correlated data per study.^27–30^

## Conclusions

In this paper, we have focused on the data analysis in a DTA systematic review. For a general overview of the DTA review process, see Leeflang *et al*.^8^ ^9^ Meta-analyses of diagnostic accuracy studies can provide answers to important clinical questions but the methods recommended are demanding. We hope our paper will provide clinicians with sufficient understanding of the terminology and methods to aid interpretation of systematic reviews and facilitate better patient care. We advise review teams to seek the support of a statistician if statistical expertise is lacking in the team. Hierarchical models can be problematic to fit, especially when there are sparse data. The models may also need to be simplified when there are few studies and meta-analysis is judged to be appropriate.^31^ The Cochrane Handbook for DTA Reviews^11^ provides guidance and is available at http://dta.cochrane.org/. The website also contains links to training materials and tutorials that can aid review authors in producing methodologically rigorous reviews. For more examples of DTA reviews on topics related to mental health, we refer readers to the Cochrane Library.

## References

[1] Naaktgeboren CA, Bertens LC, van Smeden M, et al Value of composite reference standards in diagnostic research. BMJ 2013;347:f5605 10.1136/bmj.f5605 24162938

[2] Bachmann LM, Puhan MA, ter Riet G, et al Sample sizes of studies on diagnostic accuracy: literature survey. BMJ 2006;332:1127–9. 10.1136/bmj.38793.637789.2F 16627488 PMC1459608

[3] Dahabreh IJ, Chung M, Kitsios GD, et al Comprehensive overview of methods and reporting of meta-analyses of test accuracy. Rockville, MD: Agency for Healthcare Research and Quality, 2012. 22553887

[4] Steingart KR, Sohn H, Schiller I, et al Xpert® MTB/RIF assay for pulmonary tuberculosis and rifampicin resistance in adults. Cochrane Database Syst Rev 2013;1:CD009593 10.1002/14651858.CD009593.pub2 PMC4470352 23440842

[5] Automated real-time nucleic acid amplification technology for rapid and simultaneous detection of tuberculosis and rifampicin resistance: Xpert MTB/RIF assay for the diagnosis of pulmonary and extrapulmonary TB in adults and children. Policy update. Geneva: World Health Organization, 2013. 25473701

[6] Soares-Weiser K, Takwoingi Y, Panesar SS, et al The diagnosis of food allergy: a systematic review and meta-analysis. Allergy 2014;69:76–86. 10.1111/all.12333 24329961

[7] Muraro A, Werfel T, Hoffmann-Sommergruber K, et al EAACI food allergy and anaphylaxis guidelines: diagnosis and management of food allergy. Allergy 2014;69:1008–25. 10.1111/all.12429 24909706

[8] Leeflang MM, Deeks JJ, Gatsonis C, et al., Cochrane Diagnostic Test Accuracy Working Group. Systematic reviews of diagnostic test accuracy. Ann Intern Med 2008;149:889–97. 10.7326/0003-4819-149-12-200812160-00008 19075208 PMC2956514

[9] Leeflang MM, Deeks JJ, Takwoingi Y, et al Cochrane diagnostic test accuracy reviews. Syst Rev 2013;2:82 10.1186/2046-4053-2-82 24099098 PMC3851548

[10] Quinn TJ, Fearon P, Noel-Storr AH, et al Informant Questionnaire on Cognitive Decline in the Elderly (IQCODE) for the diagnosis of dementia within community dwelling populations. Cochrane Database Syst Rev 2014;4:CD010079 10.1002/14651858.CD010079.pub2 24719028

[11] Macaskill P, Gatsonis C, Deeks JJ, et al Chapter 10: analysing and presenting results. In: Deeks JJ, Bossuyt PM, Gatsonis C Cochrane Handbook for Systematic Reviews of Diagnostic Test Accuracy Version 1.0. The Cochrane Collaboration, 2010 http://srdta.cochrane.org/, Version 1.0.

[12] Irwig L, Macaskill P, Glasziou P, et al Meta-analytic methods for diagnostic test accuracy. J Clin Epidemiol 1995;48:119–30; discussion 131–112 10.1016/0895-4356(94)00099-C 7853038

[13] Moses LE, Shapiro D, Littenberg B Combining independent studies of a diagnostic test into a summary ROC curve: data-analytic approaches and some additional considerations. Stat Med 1993;12:1293–316. 10.1002/sim.4780121403 8210827

[14] Arends LR, Hamza TH, van Houwelingen JC, et al Bivariate random effects meta-analysis of ROC curves. Med Decis Making 2008;28:621–38. 10.1177/0272989X08319957 18591542

[15] Ma X, Nie L, Cole SR, et al Statistical methods for multivariate meta-analysis of diagnostic tests: an overview and tutorial. Stat Methods Med Res Published Online First: 26 Jun 2014. 10.1177/0962280213492588 10.1177/0962280213492588 PMC3883791 23804970

[16] Reitsma JB, Glas AS, Rutjes AW, et al Bivariate analysis of sensitivity and specificity produces informative summary measures in diagnostic reviews. J Clin Epidemiol 2005;58:982–90. 10.1016/j.jclinepi.2005.02.022 16168343

[17] Chu H, Guo H, Zhou Y Bivariate random effects meta-analysis of diagnostic studies using generalized linear mixed models. Med Decis Making 2010;30:499–508. 10.1177/0272989X09353452 19959794 PMC3035476

[18] Rutter CM, Gatsonis CA A hierarchical regression approach to meta-analysis of diagnostic test accuracy evaluations. Stat Med 2001;20:2865–84. 10.1002/sim.942 11568945

[19] Harbord RM, Deeks JJ, Egger M, et al A unification of models for meta-analysis of diagnostic accuracy studies. Biostatistics 2007;8:239–51. 10.1093/biostatistics/kxl004 16698768

[20] Zwinderman AH, Bossuyt PM We should not pool diagnostic likelihood ratios in systematic reviews. Stat Med 2008;27:687–97. 10.1002/sim.2992 17611957

[21] Leeflang MM, Deeks JJ, Rutjes AW, et al Bivariate meta-analysis of predictive values of diagnostic tests can be an alternative to bivariate meta-analysis of sensitivity and specificity. J Clin Epidemiol 2012;65:1088–97. 10.1016/j.jclinepi.2012.03.006 22742916

[22] Carvalho AF, Takwoingi Y, Sales PM, et al Screening for bipolar spectrum disorders: a comprehensive meta-analysis of accuracy studies. J Affect Disord 2014;172C:337–46. 10.1016/j.jad.2014.10.024 25451435

[23] Higgins JP, Thompson SG, Deeks JJ, et al Measuring inconsistency in meta-analyses. BMJ 2003;327:557–60. 10.1136/bmj.327.7414.557 12958120 PMC192859

[24] Takwoingi Y, Leeflang MM, Deeks JJ Empirical evidence of the importance of comparative studies of diagnostic test accuracy. Ann Intern Med 2013;158: 544–54. 10.7326/0003-4819-158-7-201304020-00006 23546566

[25] Abba K, Kirkham AJ, Olliaro PL, et al Rapid diagnostic tests for diagnosing uncomplicated non-falciparum or Plasmodium vivax malaria in endemic countries. Cochrane Database Syst Rev 2014;12:CD011431 10.1002/14651858.CD011431 PMC4453861 25519857

[26] Takwoingi Y, Deeks J MetaDAS: a SAS macro for meta-analysis of diagnostic accuracy studies. User Guide Version 1.3 2010 http://dta.cochrane.org/sites/dta.cochrane.org/files/uploads/MetaDAS Readme v1.3 May 2012.pdf (accessed 11 Aug 2015).

[27] Riley RD, Ahmed I, Ensor J, et al Meta-analysis of test accuracy studies: an exploratory method for investigating the impact of missing thresholds. Syst Rev 2015;4:12 10.1186/2046-4053-4-12 25652323 PMC4417327

[28] Hamza T, Arends L, van Houwelingen H, et al Multivariate random effects meta-analysis of diagnostic tests with multiple thresholds. BMC Med Res Methodol 2009;9:73 10.1186/1471-2288-9-73 19903336 PMC2787531

[29] Dukic V, Gatsonis C Meta-analysis of diagnostic test accuracy assessment studies with varying number of thresholds. Biometrics 2003;59:936–46. 10.1111/j.0006-341X.2003.00108.x 14969472 PMC10425262

[30] Putter H, Fiocco M, Stijnen T Meta-analysis of diagnostic test accuracy studies with multiple thresholds using survival methods. Biom J 2010;52:95–110. 10.1002/bimj.200900073 19924701

[31] Takwoingi Y, Guo B, Riley RD, et al Performance of methods for meta-analysis of diagnostic test accuracy with few studies or sparse data. Stat Methods Med Res Published Online First: 26 Jun 2015, 10.1177/0962280215592269 10.1177/0962280215592269 PMC5564999 26116616

[32] Hirschfeld RM, Williams JB, Spitzer RL, et al Development and validation of a screening instrument for bipolar spectrum disorder: the Mood Disorder Questionnaire. Am J Psychiatry 2000;157:1873–5. 10.1176/appi.ajp.157.11.1873 11058490

[33] Ghaemi SN, Miller CJ, Berv DA, et al Sensitivity and specificity of a new bipolar spectrum diagnostic scale. J Affect Disord 2005;84:273–7. 10.1016/S0165-0327(03)00196-4 15708426

[34] Angst J, Adolfsson R, Benazzi F, et al The HCL-32: towards a self-assessment tool for hypomanic symptoms in outpatients. J Affect Disord 2005;88:217–33. 10.1016/j.jad.2005.05.011 16125784

